# Current Perspectives on the Molecular and Clinical Relationships between Primary Biliary Cholangitis and Hepatocellular Carcinoma

**DOI:** 10.3390/ijms25042194

**Published:** 2024-02-12

**Authors:** Annarosa Floreani, Daniela Gabbia, Sara De Martin

**Affiliations:** 1 University of Padova, 35122 Padova, Italy; annarosa.floreani@unipd.it; 2 Scientific Consultant IRCCS Negrar, 37024 Verona, Italy; 3 Department of Pharmaceutical and Pharmacological Sciences, University of Padova, 35131 Padova, Italy; daniela.gabbia@unipd.it

**Keywords:** PBC, liver cancer, HCC, risk factors, HBV, HCV, UDCA, sex difference

## Abstract

Primary biliary cholangitis (PBC) is an autoimmune liver disease characterised by the immune-mediated destruction of small and medium intrahepatic bile ducts, with variable outcomes and progression. This review summarises the state of the art regarding the risk of neoplastic progression in PBC patients, with a particular focus on the molecular alterations present in PBC and in hepatocellular carcinoma (HCC), which is the most frequent liver cancer in these patients. Major risk factors are male gender, viral infections, e.g., HBV and HCV, non-response to UDCA, and high alcohol intake, as well as some metabolic-associated factors. Overall, HCC development is significantly more frequent in patients with advanced histological stages, being related to liver cirrhosis. It seems to be of fundamental importance to unravel eventual dysfunctional molecular pathways in PBC patients that may be used as biomarkers for HCC development. In the near future, this will possibly take advantage of artificial intelligence-designed algorithms.

## 1. Introduction

Primary biliary cholangitis (PBC) is an autoimmune cholestatic chronic disease affecting the liver, characterised by a T cell-mediated destruction of small and medium intrahepatic bile ducts [1]. Its prevalence in Europe has an overall rate of 22.27 cases per 100,000 inhabitants and a pooled incidence rate of 1.7 new cases per 100,000 inhabitants per year [2], with a female predominance (1.6–4.8:1 female:male ratio). PBC may progress slowly and with a variable course over many years, leading to fibrosis and cirrhosis. These complications are more aggressive and have a worse prognosis in males than in females [3].

Many studies have evaluated the correlation between PBC and the risk of cancer development in the liver, biliary system, and extrahepatic organs. In this context, it is well known that a potentially fatal complication of PBC is the development of hepatocellular carcinoma (HCC). This cancer is the primary malignancy of the liver and has an incidence of 13 per 1000 person-years in patients with PBC and cirrhosis, which is much lower (2.7 per 1000 person-years) among PBC patients without cirrhosis [4]. HCC mostly occurs in patients with chronic liver disease, and cirrhosis represents the main risk factor independent of the liver disease aetiology [5]. Epidemiological data suggest that one-third of cirrhotic patients are at risk of developing liver cancer during their lifetime. The highest incidence of HCC has been observed in cirrhotic patients with viral aetiology (2% for HBV and 3–8% for HCV [6]) and lower in those with alcohol-related and metabolic dysfunction-associated steatohepatitis (MASH)-related cirrhosis. However, it should be stressed that metabolic-associated liver disease is becoming a more and more common HCC aetiology worldwide, and its impact may be underestimated [7]. Other less common causes of cirrhosis are autoimmune liver diseases, e.g., PBC, autoimmune hepatitis, and haemochromatosis, even though increased risk for HCC is present in these patients with respect to healthy subjects. Other important environmental and sociodemographic risk factors for HCC are cigarette smoking, ethnic differences, and exposure to aflatoxin B1 deriving from fungal contamination of foodstuffs (e.g., grains). The last risk factor is particularly relevant in some areas of Africa and Asia [8,9].

Notably, a systematic literature review analysing published data obtained from Japanese studies observed that the interval between HCC diagnosis and death in PBC patients (8.4 ± 14 months) is notably shorter than that observed in all HCC patients and in patients with autoimmune hepatitis (AIH), another autoimmune chronic liver disease [10].

The second primary liver cancer is cholangiocarcinoma (CCA), which derives from the tumoural transition of bile duct-lining epithelial cells, mainly cholangiocytes. Notably, although having a higher prevalence than other cholestatic liver diseases, such as, for example, primary sclerosing cholangitis (PSC) [11], PBC has never been associated with an increased risk of developing CCA. Accordingly, the cases of CCA are extremely rare among PBC patients (between 0.3% and 4.22%) and are mainly linked to liver cirrhosis [12].

PBC has also been associated with extrahepatic tumours, but data regarding the incidence of these diseases in PBC patients are scarce and inconclusive. The first systematic meta-analysis, including 16,300 PBC patients from several countries, showed that PBC patients had a significantly higher overall risk of developing cancer (especially HCC, as outlined above). However, no significant association was found between PBC and extrahepatic malignancies, including colorectal cancer, lung cancer, kidney cancer, oesophagus cancer, uterus cancer, cervical cancer, prostate cancer, bladder cancer, thyroid cancer, melanoma, nonmelanoma skin cancer, Hodgkin disease, and non-Hodgkin lymphoma. However, it should be underlined that this lack of correlation may be due to the extremely limited number of available studies evaluating this association [13]. In 2014, we published an epidemiological study conducted on two series of PBC patients followed up by two European centres (361 in Padova, Italy and 397 in Barcelona, Spain) [14]. The cancer incidence was compared with the standardised incidence ratio (SIR) calculated using the Cancer Registry of the Veneto Region and the Cancer Registry of Tarragona (Spain). The prevalence of cases with extrahepatic malignancy was similar in Padova (97%) and Barcelona (94%). The overall cancer incidence was similar to the expected incidence for the general population in the same geographical areas. Advanced histological stage and the presence of extrahepatic autoimmune diseases were risk factors significantly associated with the development of extrahepatic malignancy in PBC patients. A recently published multicentric cooperative study examining PBC patients admitted to Brazilian hepatology tertiary centres evaluated the frequency of HCC and extrahepatic malignancies [15]. In this cohort, cirrhosis, obesity, and azathioprine therapy were independent risk factors for HCC development, whereas Sjögren’s syndrome and psoriasis were associated with extrahepatic malignancies. These authors also suggested that pharmacotherapy with fibrates may be a protective factor toward the development of extrahepatic tumours, even if the mechanistic reasons for this observation remain to be understood.

In this review, we summarise the recent evidence regarding the risk of developing HCC in PBC patients, starting from a focus on the molecular mechanisms linking PBC pathogenesis and HCC development.

## 2. Shared Molecular Alterations in PBC and HCC

The main pathological features of PBC are immune dysregulation and abnormal bile metabolism [16], which progress to fibrosis and ultimately lead to cirrhosis and liver failure in approximately 10–20 years if not pharmacologically treated [17]. In PBC, the loss of immune tolerance against the E2 component of the pyruvate dehydrogenase complex (PDC-E2) causes the dysregulation of both innate and adaptive immunity. In turn, this results in the hyperactivation of the immune response towards the biliary epithelial cells. The increase in anti-mitochondrial autoantibodies (AMAs), a typical immune signature of PBC patients, targets the PDC-E2 in cholangiocytes, causing the apoptosis of biliary epithelial cells and thereby hampering the physiological architecture of small and medium intrahepatic bile ducts [18]. PBC patients are also characterised by a great infiltration of autoreactive CD4+ and CD8+ T cells, responsible for chronic inflammation and, ultimately, hepatic cirrhosis.

**Figure 1:**
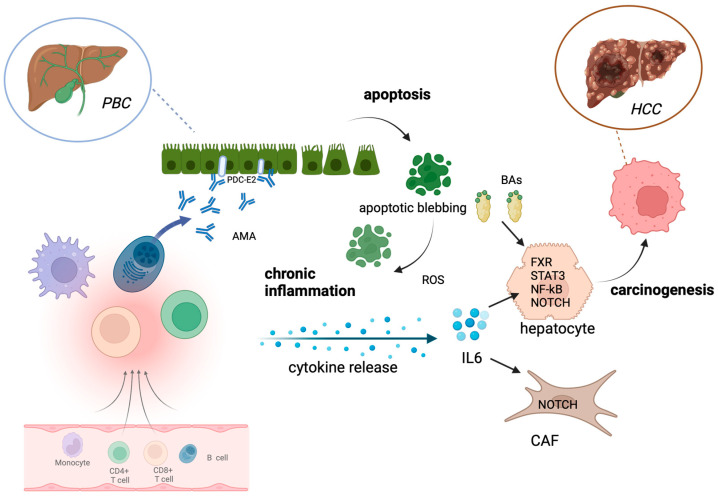
Main mechanisms involved in HCC development in PBC patients. AMA autoreactive antibodies (anti-PDC-E2) trigger cholangiocytes, inducing apoptosis and dysregulation of both innate and adaptive immunity by recruiting immune cells. This leads to an increased release of cytokines, e.g., IL6, and increased ROS production. In hepatocytes, the inflammatory microenvironment activates protumoural molecular pathways, e.g., STAT3, NF-kB, NOTCH signalling. The increased level of intrahepatic BA activates FXR and further promotes HCC development. Other parenchymal cells, such as CAFs and protumoural macrophages, are also stimulated by cytokine release, helping with HCC development. PBC, primary biliary cholangitis; AMA, anti-mitochondrial autoantibody; ROS, reactive oxygen species; IL6, interleukin 6; FXR, Farnesoid X receptor; STAT3, signal transducer and activator of transcription 3; CAF, cancer-associated fibroblast; PDC-E2, pyruvate dehydrogenase complex. Created with BioRender.com.

The molecular mechanisms favouring tumoural development in PBC patients have been only partially understood, and mechanistic studies are still warranted in this context. However, some common pathways could be recognised in PBC and HCC development (Figure 1). For example, HCC and PBC show an overlapping of hepatic dysfunction, among other inflammation. It should be noticed that when the carcinogenesis process occurs in a liver with cirrhosis, the causal relationship between the presence of an inflammatory context and the onset of the neoplastic transition in hepatic cells is difficult to establish. However, a hepatic microenvironment characterised by chronic inflammation and oxidative stress, both features of PBC patients’ livers, helps the accumulation of genetic alterations in hepatocytes [19], a phenomenon with pathological relevance in the HCC transition [20]. From a mechanistic point of view, it has been extensively demonstrated that pro-inflammatory cytokines, such as interleukin 6 (IL6), secreted by resident or recruited immune cells or their progenitors exert a stimulatory action on the growth of both normal and neoplastic hepatocytes [21]. Interestingly, IL6 and other IL17-related cytokines are upregulated in PBC patients [17]. Mechanistic details revealed that IL6 induces cell proliferation by a STAT3-dependent mechanism and by the activation and direct interaction with the p65 subunit of NF-kB, a transcription factor known to exert a complex panel of pro-inflammatory actions [22]. Notably, IL6 hepatic secretion is differently regulated in men and women due to estrogens [23], which are able to counteract IL6 secretion by Kupffer cells in the liver and decrease tumour growth [24]. These observations came from a preclinical in vivo study, which also demonstrated that IL6 ablation abolished the observed sex differences in hepatocarcinogenesis, suggesting IL6 as a pharmacological target for HCC [25]. This gender difference in the production of inflammatory cytokines may at least partially explain the fact that male sex is a risk factor for HCC development, although PBC is more frequent in females. Another signalling pathway known to be altered in PBC patients and related to STAT3 is NOTCH. Proteins of this molecular pathway are overexpressed in PBC [26]. In particular, an immunohistochemical study demonstrated that NOTCH1 expression was increased in the reactive ductuli of cirrhotic PBC samples [27]. Interestingly, a gain in NOTCH signalling has also been associated with hepatocarcinogenesis, notably in HCC. The pioneering study of Villanueva and collaborators [28] paved the route for understanding the role of this molecular pathway in HCC. These authors, besides confirming that NOTCH signalling is activated in human HCC samples, observed that it promotes the formation of liver tumours in mice. More recent evidence further investigated the role of NOTCH in HCC development. First, it is clear that NOTCH acts as a cancer promoter since its target genes are transcription factors that control numerous tumour-related cellular processes, including proliferation, differentiation, and apoptosis [29]. A more complex picture is depicted when considering not only cancer cells but also the tumour microenvironment (TME). Notably, NOTCH signalling agonists promote the activation of macrophages [30], whose role in the TME is extensively described [31]. Other cells present in the TME, playing a peculiar role in HCC progression, are cancer-associated fibroblasts (CAFs). Besides modulating the biological activities of HCC [32], their presence has been linked to HCC cell growth and metastasis. HCC cells stimulate the proliferation of CAFs, which can secrete high amounts of IL6. As outlined before, the fundamental role of IL6 in HCC progression is well established. This study demonstrated that, when secreted by CAFs, it facilitates characteristics of staminality in HCC cells, and this occurs by activating the NOTCH signalling pathway by the phosphorylation of STAT3 [33].

Another interesting feature of PBC, which can also be linked to HCC development, is the composition of the pool of bile acids (BAs) in patients affected by this disease. To understand BA relevance in this context, it is worth mentioning the two drugs approved for PBC therapy, i.e., ursodeoxycholic acid (UDCA) and obeticholic acid (OCA), which are the first- and second-line treatments for PBC, respectively [34]. These are the two drugs approved for PBC treatment, although some authors have suggested that a single drug or a single mechanism is probably not completely effective in stopping disease progression and avoiding cirrhosis and other complications and advise of the need for combinatorial approaches targeting multiple mechanisms [17].

**Figure 2:**
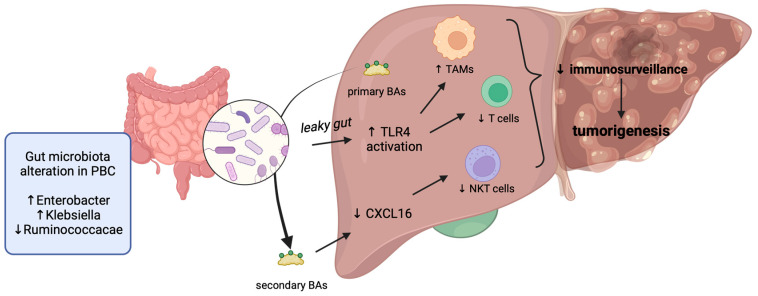
Increased conversion of primary into secondary bile acids is affected by gut microbiota dysregulation in PBC patients. This alteration leads to leaky gut and alters hepatic immune cells, thereby reducing immunosurveillance and promoting tumourigenesis. BAs, bile acids; TLR4, toll-like receptor 4; CXCL16, Chemokine (C-X-C motif) ligand 16; NKT, natural killer T cells; TAM, tumour-associated macrophages. Created with BioRender.com.

UDCA is a hydrophilic, non-cytotoxic BA, usually accounting for less than 5% of the BA pool. UDCA has multiple mechanisms of action, including the replacement of endogenous cytotoxic BAs, such as chenodeoxycholic acid (CDCA) and deoxycholic acid (DCA) [35]. Notably, men, who are more prone to developing PBC complications, have a higher total concentration of BAs than women. Another finding is that PBC patients display a different BA conjugation pattern than healthy subjects, having higher rates of taurine BA conjugation. Notably, the conjugative agent taurine can reduce the hepatotoxicity of hydrophobic BAs more than glycine. This is probably an adaptive mechanism exploited by PBC patients [36]. Taken together, these observations led to the interesting hypothesis that PBC patients not responding to UDCA treatment might have higher plasma concentrations of BAs, particularly of the cytotoxic CDCA, that cannot be efficiently replaced by UDCA or a reduced capacity of taurine conjugation [37]. Thus, the increased amount of BAs in the livers of these patients probably favours cancer development. The mechanism by which hydrophobic BAs prompt liver carcinogenesis has been deeply investigated by numerous in vitro and in vivo preclinical studies. A collaborative action of different BAs, all characterised by high hydrophobicity, has been observed in downregulating tumour suppressor genes like CEBPα, thereby helping the development of liver cancer [38]. Altered BA levels, besides leading to metabolic dysfunction [39], can induce senescence, resistance to apoptosis, and hyper-proliferation [40], all hallmarks of the neoplastic transition. BAs can exert a significant cytotoxic effect when they accumulate inside the liver at high concentrations because of their direct cytolytic action [41]. Furthermore, it is well known that Farnesoid X receptor (FXR), a nuclear receptor activated by CDCA, is involved in the modulation of cancer development [24,42,43]. Notably, FXR and STAT3 signalling act together in liver carcinogenesis. In fact, the persistent activation of STAT3 is present in the livers of FXR knockout mice [44]. The mechanism relies on the upregulation of IL6 due to the high BA amount typical of these mice. In fact, BAs are strong STAT3 inducers [45], and one of the target genes of FXR, the suppressor of cytokine signalling 3 (SOCS3) [46], is a feedback inhibitor of STAT3. Taken together, all these molecular dysregulations collectively lead to STAT3 constitutive activation [44]. Other pharmacological data support the pivotal role of FXR in HCC development. OCA, which is an FXR agonist, reduces the proliferation and metastatic properties of HCC cells, and this is due to the inhibition of the IL6/STAT3 signalling [47]. The role of BAs in HCC development is further supported by the observation that the relative amount of primary and conjugated BAs is altered in preclinical models and patients with HCC. The increased conversion of primary to secondary conjugated BAs has been linked to the alteration of gut microbiota [41,48,49]. Notably, these microbiota-associated dysregulations of BAs have been correlated to immune-related alterations in the liver, for example, downregulation of the Chemokine (C-X-C motif) ligand 16 (CXCL16), a chemotactic cytokine with a peculiar role in cancer [50]. Interestingly, the CXCL16 downregulation reduces hepatic CXCR6+ natural killer T (NKT) cells, immune cells involved in immune surveillance [40,51]. Recently, many studies have observed that the gut microbiota is significantly altered in PBC patients, and dysbiosis could act as a promoter of HCC development [49]. Moreover, the increased bacterial abundance observed in the hepatic tissue of cirrhotic patients due to the so-called leaky gut induces transcriptional changes, leading to the activation of fibro-inflammatory pathways and the modulation of the hepatic inflammatory microenvironment towards cancer cell immune escape and promoting HCC development (Figure 2). This is due to alteration in toll-like receptor 4 (TLR4) signalling pathways, which lead to a switch of tumour-associated macrophages toward the protumoural M2-like phenotype, as well as to a reduction of T cell-mediated immunity [52]. An increased abundance of some specific bacterial strains has been observed in PBC, e.g., those of Enterobacter and Klebsiella, as well as a decreased abundance of *Bacteroidetes* spp. and Ruminococcaceae. Some of these dysfunctional alterations have also been observed in other chronic liver diseases and in HCC patients, suggesting that correlation between specific gut microbiota profiles and systemic inflammation could promote hepatocarcinogenesis [53]. In this context, it is reasonable to hypothesise that the alteration of gut microbiota may be involved in HCC development in PBC patients.

Genome-wide association studies (GWAS) in different cohorts of PBC patients identified strong SNP associations located in the human leukocyte antigen (HLA) class II region predisposing to PBC risks [54,55,56,57,58,59]. A meta-analysis of eight different studies has suggested an association between the HLA-DRB1 allele polymorphisms HLA-DRB1*07 and HLA-DRB1*12 and the risk of HCC, while HLA-DRB1*07, HLA- DRB1*12, and HLA-DRB1*15 alleles were associated with significantly increased risks of HCC in Asian populations [60]. Another publication analysing 12 case-control studies (2030 HCC patients and 2817 relevant controls) confirmed that HLA-DRB1*12 and HLA-DRB1*14 are risk factors for HCC development, while it was observed that HLA-DRB1*1 and HLA-DRB1*11 are protective factors [61]. A recent study by Khor et al. on Japanese PBC patients identified HLA-DPB1*05:01:01 as associated with HCC [54].

Other emerging candidate biomarkers for PBC are circulating microRNAs (miRNAs), which are extremely stable, highly conserved, non-coding small RNAs post-transcriptionally regulating gene expression [62]. A study by Tan and colleagues observed that serum levels of miR-122-5p are elevated in PBC patients and suggested that a panel of three miRNAs (miR-122-5p, miR-141-3p, and miR-26b-5p) could be a more sensitive and specific marker than ALP and ANA to diagnose PBC. miRNA-122 regulates many cell functions, among others, lipid metabolism, cell differentiation, acetaminophen toxicity, liver fibrosis in innate immunity, and may play a role in the proliferation and apoptosis of intrahepatic bile duct cells [63]. The expression of this miRNA has also recently been investigated in HCC patients, and surprisingly, its decreased expression was found to be associated with metastasis in HCV-negative HCC [64]. Thus, miRNA-122 may deserve further research in this field to ascertain whether and how it could be useful for the identification of PBC patients prone to HCC development, also in light of the fact that differences in miRNA expression may be affected by geographic differences [65].

## 3. Hepatocellular Carcinoma in PBC Patients

HCC represents at least 75% of primary liver malignancies, being one of the main cancer-related causes of death worldwide, with poor prognosis in the case of advanced stage [20,66]. Viral hepatitis, e.g., HBV and HCV, alcoholic liver disease, and metabolic dysfunction-associated steatotic liver disease (MASLD), are considered the major risk factors for HCC. A limited number of studies have been focused on assessing the risk of HCC development in PBC patients. The main studies investigating the incidence of HCC cases in PBC patients are reported in Table 1.

A meta-analysis evaluating the incidence of HCC in PBC has been performed, including 29 studies for a total of 22,615 patients [84]. The pooled incidence ratio was 4.17 per 1000 patient-years. On subgroup analysis in patients with cirrhosis, the incidence was 15.70 per 1000 person-years. The incidence rate was higher in men (9.82 per 1000 person-years) than in women (3.82 per 1000 person-years). These findings confirm the high risk of developing HCC for patients with PBC. However, the risk of HCC was low in patients without cirrhosis; moreover, the treatment with UDCA did not reduce the overall risk of HCC, nor in patients with PBC associated with cirrhotic stage.

### 3.1. Risk Factors for HCC in PBC Patients

The risk factors that have been correlated to an increased incidence of HCC in PBC patients have been and are currently investigated. Eleven studies reported a relationship between HCC and PBC severity; all of them clearly indicate that HCC arises in advanced histological stages [15,68,69,70,72,76,78,79,80,81]. This behaviour is most commonly observed in all types of liver disease, with the exception of MASLD, in which it seems that the pre-cirrhotic stage might confer an increased risk of HCC, independent of cirrhosis [85]. These data also confirm our previous observation, i.e., that the relative risk for HCC in female patients with PBC in the cirrhotic stage is similar to that of female patients with cirrhosis of different aetiologies [86].

The most impressive data regarding the analysis of risk factors for HCC shows the association with male gender and the lack of response to UDCA. In particular, the selection of studies listed in Table 2 ranked on the basis of the percentage of HCC cases in each PBC patient cohort, besides confirming the pivotal role of histological stage severity, underlines that male gender is definitely a fundamental risk factor for the neoplastic transition of PBC patients. Male sex has, in fact, been recognised as a risk factor in most of the studies (54%, 7 studies out of 13) and is the one that has been most commonly identified. The second risk factor in terms of frequency is the unresponsiveness to UDCA treatment (23% of the studies, 3 out of 13), together with the advanced age of patients, which has been identified as a risk factor per se in two of the considered studies, while in one of them, the authors indicate the “age at diagnosis” [72]. One study from Japan [72] and one from China [80] found an association between HCC and a history of blood transfusion and a history of HBV infection, respectively. Similarly, in our previous study, coinfection with HCV infection emerged in the multivariate analysis as an independent risk factor for HCC [68]. These studies are in favour of an important co-factor for malignancy represented by hepatitis viruses.

Recently, the analysis of the Italian Liver Cancer Registry identified 80 cases of PBC with HCC after the year 2000 [87]. The median age was 71 years, and 50% were males; cirrhosis was present in 86.3% of cases. In general, risk factors indicate that patients with PBC, similarly to those with other autoimmune liver disease, have a moderate risk for HCC [88].

A detailed description of the main risk factors that have been associated with HCC development in PBC patients is reported below.

#### 3.1.1. Male Gender

It is known that HCC incidence and mortality rates are 2–5 times higher in men than in women in different areas [89]. In general, oestrogens can protect hepatocytes from malignant transformation to HCC through the downregulation of IL6 release from Kupffer cells. Thus, HCC is more common in male patients with PBC than in females, and this sex-related difference may be partially due to the lack of oestrogen protective effect [23,90]. Moreover, some X-chromosome-located or Y-chromosome-located genes and sex hormone-related pathways have been suggested to be involved in hepatocarcinogenesis [91,92]. A recent study has demonstrated that CYP39A1, a liver-specific autosomal gene that has a female-preferential expression, strongly suppressed HCC development and resulted in a dramatic downregulation in over 90% of HCC patients [93]. Its inhibitory activity on hepatocarcinogenesis is due to the C-terminal region that blocks the transcriptional activation activity of c-Myc, and females seem to be protected due to a higher expression of this CYP isoform. Some molecular pathways involved in HCC severity are also affected by sex, namely PI3K/AKT/mTOR, Wnt/β-catenin, and TGF-β [94]. PI3K/AKT/mTOR is widely studied due to its involvement in the regulation of cell growth and proliferation, and its hyperactivation is related to worse HCC prognosis and progression. Male HCC patients displayed increased activation of this pathway with respect to females, contributing to the more aggressive features of HCC observed in this sex [95]. Interestingly, oestrogens seem to inhibit this pathway, conferring potential protection for women. Aberrant activation of Wnt/β-catenin signalling also significantly affects HCC development, progression, and clinical-driving stemness and metabolic reprogramming [96]. The oestrogen receptor 1-mediated inhibition of this pathway contributes to the protection against HCC in women [97]. At variance, testosterone can promote Wnt/β-catenin signalling, potentially contributing to increased HCC risk in men. TGF-β, a cytokine displaying a dual role in HCC, can suppress tumourigenesis at early stages, while it switches to a protumourigenic activity in late stages [98]. Its expression is strongly affected by sex and sex hormones that are known to exert a dual opposite hepatoprotective and hepatotoxic role [99].

Another point that should be stressed is that males, more than females, can have other co-factors of malignancy, including alcohol consumption, smoking, and hepatitis viruses. Moreover, a history of blood transfusions may indicate a risk for HBV or HCV transmission. In a case-control study performed in China in 52 patients with HCC in PBC (36 females and 16 males), males were more likely than females to have a history of blood transfusions, alcohol consumption, smoking, and a family history of malignancy [81].

#### 3.1.2. Hepatitis Viruses

Chronic hepatitis B virus (HBV) infection is still a main risk factor for HCC in the world since various direct and indirect mechanisms increase the risk of developing HCC with or without an underlying liver cirrhosis and promote hepatocarcinogenesis [100]. Watanabe et al. reported that past HBV infection is an important factor associated with HCC also in PBC that is likely to be attributable to the higher rates of blood transfusion in these populations of patients [10]. The risk is not eliminated by viral suppression due to HBV-DNA being integrated into the human genome. The combination of viral and host factors has synergic effects on HCC development, particularly the patient’s gender, type 2 diabetes, metabolic syndrome, and HBV core mutations [101].

Chronic hepatitis C virus (HCV) is another main risk factor for HCC. Viral eradication has reduced the incidence of HCC development, even though the risk of HCC development could not be completely eliminated beyond 10 years of sustained virological response. Thus, HCC incidence is very low but still remains [102]. Both PBC and HCV infection display chronic inflammation during progression to cirrhosis. HCV infection was a risk factor for the development of hepatocellular carcinoma (HCC) in a group overlapping PBC and HCV [103]. Indeed, HCV infection is likely to aggravate cirrhosis in PBC patients, probably due to the synergic effect of the two pathologies: PBC leads to bile duct destruction on one side, and HCV leads to hepatocyte and parenchyma injury on the other side [104]. PBC patients with concomitant HCV infection are characterised by a peculiar biochemical profile characterised by poor values of liver markers, e.g., albumin, and this comorbidity is a risk factor for the development of more severe liver damage [104].

#### 3.1.3. Lack of UDCA Response

The unresponsiveness to UDCA treatment emerged in the studies by Kuiper [105] and by Trivedi [79]. The latter study included a uniquely powered, internationally representative cohort that observed that a 12-month biochemical non-response according to Paris-I criteria is significantly associated with risk of HCC development in the future in patients with early-stage or advanced disease and also when restricting the analysis to only male patients. This study proposed a risk stratification based on a 12-month biochemical non-response that may be relevant to patient care and the development of new therapies [79].

Binu et al. analysed PBC progression in a cohort of subjects with compensated PBC cirrhosis at higher risk for clinical events, obtaining contrasting results with respect to those reported by the study of Trivedi et al. that showed a protective role of UDCA response towards HCC development, particularly greater in non-cirrhotic patients than cirrhotic ones [106]. This discrepancy has been explained by the authors by a reduction of the benefit of UDCA response due to a male-predominant cohort having an elevated risk of HCC at baseline. Likewise, in another study of cirrhotic PBC patients, the pooled incidence of HCC in patients receiving UDCA treatment was similar to those without therapy [84].

Therefore, it remains to be completely understood to what extent UDCA treatment lowers the risk of HCC development in PBC patients; thus, further investigations are advised. These observations also deserve another consideration, that is, the feasibility of the cost-effective benefit of performing an HCC screening in patients with PBC at the cirrhotic stage, mainly in male non-responders to UDCA.

#### 3.1.4. Alcohol Intake

It is well known that alcohol is a potent factor for carcinogenesis [107]. A specific association exists between alcohol and tumours of the digestive tract, including the liver. Ethanol per se is not mutagenic, but acetaldehyde, which is a product of its metabolism, is carcinogenic and mutagenic by binding to DNA and proteins [108]. A genetic predisposition in terms of ALDH-2 variants may amplify the susceptibility to carcinogenesis; indeed, East Asian populations, which have the highest prevalence of the ALDH-2 variant, show an association with upper aero-digestive tumours [109]. However, activation of the immune system has a pivotal role in keeping cancer under control and can facilitate cancer progression. Alcohol can modulate the immune response in terms of immune suppression and activation of mechanisms of growth of cancer and progression [110].

A study by Zhang and collaborators analysed HCC incidence and characteristics in a cohort of PBC-associated HCC patients (PBC patients with HCC and 77 matched controls without HCC recruited at Beijing 302 Hospital during the period January 2002–December 2013) [81]. Among other risk factors, they also assessed the history of alcohol intake (considered to be alcohol consumption at least once per week for at least 1 year without alcoholic hepatitis diagnosis), observing that it is independently associated with HCC development in Chinese patients with PBC. At variance, in the study of Cavazza et al. on two European cohorts of PBC patients, no significant association between alcohol (defined as alcohol consumption >40 g/day) and HCC was found in PBC patients [69].

#### 3.1.5. Metabolic and Age-Related Risk Factors

Notably, the study by Zhang et al. in China found that BMI ≥ 25 Kg/m^2^ was significantly associated with HCC in PBC [81]. This finding confirmed the study of Hindi et al. on 49 well-characterised AMA-positive PBC patients that observed that MASH and BMI ≥ 25 were associated with severe biliary duct damage and fibrosis [111].

Another study analysing patients with type 2 diabetes mellitus (T2DM) and HCC from different aetiologies who registered for liver transplantation observed that T2DM is a risk factor for HCC development. Intriguingly, T2DM does not represent an additional risk factor for PBC patients [112].

A study assessing throughout 20 years the incidence, risk factors, and clinical features of HCC in a cohort of 1865 well-defined Chinese PBC patients observed that age >54 years and co-existence of T2DM were independently associated with HCC development [80].

## 4. HCC Screening and Treatment in PBC Patients

According to current international guidelines, HCC screening should be performed, irrespective of the aetiology, in all cirrhotic patients with Child–Pugh A and B and in patients with Child–Pugh C awaiting liver transplantation [113,114,115], and no guidelines suggest specific or additional screening for PBC patients. Abdominal ultrasound in cirrhotic patients is the recommended method for liver cancer screening and may often successfully be used to detect one of the common complications of liver cirrhosis—portal hypertension—through the evaluation of some indirect signs, e.g., splenomegaly and portal vein diameter [116]. Ultrasound with alpha-fetoprotein (AFP) determination every six months is suggested anyway, as screening in PBC has demonstrated a significantly increased sensitivity in the early detection of HCC in patients with cirrhosis [117]. Hypoalbuminemia, thrombocytopenia, and portal hypertension, three signs of advanced PBC, are all risk factors for the development of HCC, thus supporting the HCC screening according to guidelines. A case report described the case of a 71-year-old female with overlapping AIH and PBC, with a slightly increased AFP level, and without chronic viral hepatitis and oesophageal varices or alcohol intake who presented an atypical aspect of a subcapsular hypoechoic nodule in the absence of other risk factors, except for liver cirrhosis [118]. The nodule could have been a regenerative nodule at the first diagnosis if it were not for the fact that its dimensions increased in time; thus, it was finally diagnosed as HCC BCLC Stage A. Thus, this report further supports the need for proper surveillance every 6 months, including abdominal ultrasound and AFP levels, for cirrhotic patients in order to diagnose HCC early.

Compared to HCC deriving from other chronic liver diseases, PBC-associated HCC has a poor prognosis with a median survival of 36 months without treatment. Recently, thanks to early diagnosis and improved treatment, the survival time has increased by years. Regarding the best clinical option, liver transplantation has granted the highest survival rate in cirrhotic patients with severe liver damage or who progressed to liver failure [4], even though the indication for transplantation in PBC patients is the same as in any other form of chronic liver disease, with HCC and HCC as a quite rare indication for transplantation [119,120]. Moreover, pretransplant assessment and inclusion criteria for the waiting list may vary between transplant teams, and the optimal approach is generally continuously under re-evaluation and changing in each transplant centre [121].

## 5. Future Perspectives and Conclusions

The incidence of PBC is still rising worldwide. Although it seems that this incidence has reached a plateau in North America and Europe, it is still increasing in the Asia-Pacific region, probably due to the increased reporting rate of new diagnoses [122]. On this basis, we also expect that the incidence of HCC in PBC patients will increase in the near future. On the other hand, the association between metabolic syndrome, diabetes, and obesity and HCC in patients with MASLD has been demonstrated [123,124,125,126]. As one-third of patients with PBC actually have metabolic syndrome [127], it is reasonable to hypothesise that an increased incidence of HCC associated with PBC will develop in the future.

The key research areas in unravelling the molecular and clinical relationships between PBC and HCC are the identification of (i) novel biomarkers for the early diagnosis of PBC patients, particularly those at risk of HCC development, and (ii) novel therapeutic targets able to prevent PBC–HCC progression. Indeed, it is of fundamental importance to find some pathological, biological, and genetic features in HCC patients arising from PBC that may be used as biomarkers or exploited therapeutically to target the healthy–malignant transition. This aim can be pursued with a better understanding of the mechanisms leading to HCC development in PBC patients since the molecular drivers of this transition are only partially understood and described. To this purpose, artificial intelligence (AI) may be of help in the construction of algorithms predictive for HCC development in PBC patients. Some recent computational search algorithms and machine learning (ML) and deep learning (DL) models have been set up to help in HCC risk prediction, diagnosis, and prognostication [128]. Despite this promising and fascinating scenario, the standardisation of AI data is still required to obtain satisfactory results in terms of generalisability and interpretability. However, in the future, AI is likely to produce great advances in the prediction of PBC-related HCC occurrence.

In conclusion, even though many risk factors are predictive of HCC development in PBC patients, the molecular mechanisms helping HCC onset in these patients remain to be fully understood. Therefore, further studies are encouraged to improve the early identification of populations with high risk to be checked by periodic screening for HCC.

## Figures and Tables

**Table 1:** Main studies reporting the number of HCC cases in cohorts of PBC patients.

Study	Country	Study Period	n. PBC Patients	n. HCC Cases
Goldacre MJ [67]	UK	1963–1999	424	8
Floreani A [68]	Italy	1973–1996	175	4
Cavazza A [69]	Spain	NA	389	13
Cavazza A [69]	Italy	1973–2007	327	11
Jones D [70]	UK	1975–1995	667	16
Nijhawan P [71]	USA	1976–1985	1692	12
Shibuya A [72]	Japan	1980–1998	396	14
Howel D [73]	UK	1987–1994	769	7
Jackson H [74]	UK	1987–2002	930	7
Su C-W [75]	Taiwan	1985–2006	96	5
Deutsch M [76]	Greece	1987–2005	212	8
Kuiper EM [77]	The Netherland	1990–2007	375	9
Harada H [78]	Japan	1980–2009	2946	71
Trivedi PJ [79]	Global PBC	1959–2012	4565	123
Rong G [80]	China	1994–2014	1865	70
Braga MH [15]	Brasil	1992–2020	752	20
Zhang X-X [81]	China	2002–2013	1255	52
Cheng J-S [82]	Taiwan	2002–2015	2737	146
Boonstra K [83]	The Netherland	2008–2011	992	7

**Table 2:** Selected studies reporting the percentage of cases, histological stage, and/or identified risk factors for HCC in cohorts of PBC patients.

Study	% HCC Cases/PBC Patients	Histological Stage	Risk Factors
Cheng J-S [82]	5.33	NA	Male sex
Zhang X-X [81]	4.14	100% cirrhosis	BMI ≥, alcohol intake
Deutsch M [76]	3.77	IV	Advanced histological stage
Rong G [80]	3.75	80% with III/IV stage	Advanced age, male sex, co-existence of diabetes, history of HBV infection
Shibuya A [72]	3.54	III-IV	Male gender, age at diagnosis, history of blood transfusion
Cavazza A [69]	3.36	III-IV	Male gender
Trivedi PJ [79]	2.69	42% with advanced disease	Advanced age, male sex, thrombocytopenia at 12 months, non-response to UDCA
Braga MH [15]	2.66	95% with cirrhosis	Cirrhosis, obesity, prior azathioprine use
Harada H [78]	2.41	10 I/17 II/14 III/8 IV	Male sex, advanced histological stage (in females)
Kuiper EM [77]	2.40		Lack of response to UDCA
Jones D [70]	2.40	IV	Male gender
Floreani A [68]	2.29	IV	HCV, smoking
Jackson H [74]	0.75	NA	UDCA seems protective

## Data Availability

Not applicable.
